# Recorded Adverse Events and Patient-Reported Outcomes After Intra-articular Extracellular-Vesicle Product Administration for Knee Osteoarthritis: A Retrospective Single-Practice Case Series

**DOI:** 10.7759/cureus.117960

**Published:** 2026-10-15

**Authors:** Jonathan Courtney, Cassandra Cortese, Kaitlyn Hollman

**Affiliations:** 1 Surgery, Florida Atlantic University Charles E. Schmidt College of Medicine, Boca Raton, USA; 2 Adult Reconstruction/Sports Medicine, Orthopedic Surgery Associates, Boca Raton, USA

**Keywords:** exosomes, extracellular vesicles, intra-articular injections, knee osteoarthritis, koos-jr, mesenchymal stem cells (mscs), orthobiologics

## Abstract

Background

Exosome-based products are being used for symptomatic knee osteoarthritis (OA), although human efficacy data remain limited and no extracellular vesicle product is approved by the United States Food and Drug Administration for intra-articular use. This study evaluated patient-reported outcomes, subsequent interventions, conversion to total knee arthroplasty (TKA), and recorded adverse events following intra-articular treatment.

Objective

The aim of the study was to compare pre-injection Knee injury and Osteoarthritis Outcome Score for Joint Replacement (KOOS-JR) scores with scores obtained at six weeks and at the latest follow-up, and to document subsequent conversion to TKA.

Study design

This retrospective case series provides Level IV evidence.

Methods

Consecutive intra-articular knee treatments administered between December 2024 and August 2026 were retrospectively reviewed. Patients received either Patient Pure X (PPX™), an autologous platelet-derived extracellular-vesicle preparation, or DayZero™, an allogeneic perinatal tissue-derived extracellular-vesicle preparation manufactured by ZEO ScientifiX. For the primary patient-level analysis, one eligible index knee per patient was included to preserve independence of observations; laterality-ambiguous bilateral KOOS-JR observations were excluded from knee-specific patient-reported outcome analyses. Primary outcomes were paired changes in KOOS-JR from baseline to six weeks and latest follow-up. Secondary outcomes included subsequent nonsurgical interventions, subsequent TKA, and documented administration-related adverse events. Paired t-tests and Wilcoxon signed-rank tests were used.

Results

Fifty-one treated knees in 47 patients were included. Mean age was 75 years (range, 44-94 years); 28 patients were women, and 19 were men. In the primary patient-level analysis, 33 patients had complete, laterality-attributable paired baseline and six-week KOOS-JR data. Mean KOOS-JR increased from 60.0 to 78.5, a mean increase of 18.5 points (95% CI, 13.2-23.8; p<0.001); 30 of 33 patients (90.9%) had higher scores. At the latest follow-up, 36 patients had complete paired data; mean KOOS-JR increased from 60.6 to 77.4, a mean increase of 16.8 points (95% CI, 11.4-22.3; p<0.001), with 30 of 36 patients (83.3%) having higher scores. Three of 47 patients subsequently underwent TKA. No administration-related adverse events were documented in the available practice records; however, adverse-event ascertainment was retrospective.

Conclusions

In this retrospective single-practice case series, mean KOOS-JR was 18.5 points higher at six weeks than at baseline among 33 patients with complete, laterality-attributable paired data following intra-articular administration of extracellular-vesicle products. Later outcome assessments were limited by nonuniform follow-up and subsequent treatments. Three of 47 patients subsequently underwent TKA. No administration-related adverse events were documented in the available practice records; however, retrospective ascertainment and the limited sample size preclude conclusions regarding safety or tolerability. These descriptive findings do not establish efficacy, clinical importance, comparative effectiveness, structural disease modification, or long-term benefit. Prospective controlled studies using well-characterized products, standardized follow-up, prespecified management of subsequent interventions, and active adverse-event surveillance are warranted.

## Introduction

Knee osteoarthritis (OA) is a leading cause of pain, functional limitation, and disability. Management is typically progressive and may include patient education, activity modification, exercise, weight management, oral or topical analgesic medication, intra-articular corticosteroid injection, hyaluronic acid injection, platelet-rich plasma (PRP), and, for end-stage disease with persistent symptoms, total knee arthroplasty (TKA). Contemporary guidelines support exercise and selected established therapies, although recommendations concerning injectable treatments vary because of heterogeneity in products, protocols, patient selection, and outcome measures [1,2].

Extracellular vesicles are membrane-bound particles released by cells that may carry proteins, lipids, and nucleic acids between cells. Exosomes are generally considered a subtype of small extracellular vesicles. However, rigorous identification requires defined biochemical, biophysical, and cell-of-origin characterization [3].

Preclinical studies suggest that mesenchymal stromal cell-derived extracellular vesicles may modulate inflammation, influence chondrocyte activity, and promote cartilage homeostasis [4-6]. These findings have generated interest in extracellular-vesicle and exosome-based treatments as potentially cell-free orthobiologic therapies for OA. However, these preclinical findings do not establish clinical efficacy in humans, and translation into routine clinical care remains preliminary. 

Although human clinical evidence regarding intra-articular extracellular-vesicle products remains limited, a randomized, triple-blind, placebo-controlled trial by Bolandnazar et al. evaluated placental mesenchymal stromal cell-derived extracellular vesicles in 29 patients with bilateral knee OA [7]. Each patient received an extracellular-vesicle injection in one knee and saline in the contralateral knee. At two and six months, no statistically significant differences were observed between treatment and placebo groups in pain, functional outcomes, or MRI findings. These findings highlight the uncertainty surrounding the clinical efficacy of extracellular-vesicle treatments and underscore the importance of controlled studies in distinguishing treatment-specific effects from placebo responses and natural symptom variability. Furthermore, no exosome product is currently approved by the United States Food and Drug Administration for treating orthopedic conditions [8].

Two distinct extracellular-vesicle products were used during the study period. Patient Pure X (PPX™; ZEO ScientifiX, Davie, Florida) was an autologous extracellular-vesicle concentrate derived from the patient’s own platelet-containing blood fraction, whereas DayZero™ (ZEO ScientifiX) was an allogeneic extracellular-vesicle product derived from perinatal tissue. Manufacturer information describes PPX™ as a platelet-derived autologous extracellular-vesicle concentrate and DayZero™ as an allogeneic, perinatal tissue-derived extracellular-vesicle preparation [9]. Of the 51 treated knees, 7 (13.7%) received PPX™ and 44 (86.3%) received DayZero™. Product selection was based on patient preference.

Each treatment consisted of a 1-mL intra-articular injection containing the extracellular-vesicle preparation. All injections were administered by the senior author (JC) in the office under sterile conditions using an anterolateral approach to the knee. Products were received on dry ice and immediately transferred to a non-defrosting freezer for storage until use. Each specimen was accompanied by a manufacturer-provided lot number and certificate of analysis, and the corresponding lot number was recorded in the patient's medical record.

Because PPX™ and DayZero™ differ in biologic source and preparation, they were not considered biologically interchangeable. They were pooled for the primary descriptive analysis because the objective of this retrospective study was to characterize clinical outcomes following extracellular-vesicle treatment within a single practice rather than to establish comparative effectiveness between products. Detailed independent biochemical characterization of individual preparations was not available in the retrospective clinical dataset.

Patient-reported outcome measures provide an important method of quantifying the effect of treatment on pain, symptoms, and function. The Knee injury and Osteoarthritis Outcome Score for Joint Replacement (KOOS-JR) is a validated seven-item instrument that produces a single score representing overall knee health [10]. Scores range from 0 to 100, with higher values indicating better knee health.

The primary objective of this retrospective study was to describe the paired change in KOOS-JR from baseline to six weeks following intra-articular extracellular-vesicle treatment for symptomatic knee OA. Secondary objectives were to describe KOOS-JR at the latest available follow-up, subsequent nonsurgical interventions, conversion to TKA, and documented administration-related adverse events.

## Materials and methods

Study design/treatment protocol

Patients received one of two intra-articular extracellular-vesicle products manufactured by ZEO ScientifiX (Davie, Florida): PPX™, an autologous platelet-derived extracellular-vesicle preparation, or DayZero™, an allogeneic perinatal tissue-derived extracellular-vesicle preparation. Of the 51 treated knees, 7 (13.7%) received PPX™ and 44 (86.3%) received DayZero™. Product selection was based on patient preference rather than clinician assignment or disease severity. Forty-three patients underwent treatment of one knee and four patients underwent treatment of both knees.

Each treated knee received a single 1-mL intra-articular injection of the selected extracellular-vesicle preparation. All injections were performed by the senior author (JC) in the office using a 25-gauge needle and a landmark-guided anterolateral approach under sterile conditions. No local anesthetic, corticosteroid, saline, or other substance was co-injected.

Products were shipped on dry ice and immediately transferred upon receipt to a non-defrosting freezer for storage. Before administration, each specimen was thawed at room temperature for approximately 30 minutes. Each specimen was accompanied by a manufacturer-provided lot number and certificate of analysis, and the corresponding lot number was recorded in the patient's medical record.

Patients had no post-injection activity restrictions and were instructed to use ice as needed for swelling and to avoid nonsteroidal anti-inflammatory drugs for two weeks. Additional treatment modalities for the treated knee were not offered for three months following injection. Subsequent nonsurgical interventions occurring thereafter were recorded as secondary outcomes.

PPX™ was an autologous extracellular-vesicle concentrate produced from the patient's own blood. According to the manufacturer, the processing method removes red blood cells and leukocytes while concentrating a platelet-derived extracellular-vesicle fraction containing proteins, growth factors, and microRNAs. Following processing, the preparation was returned for administration to the same patient from whom the blood specimen was obtained [9].

DayZero™ was an allogeneic, perinatal tissue-derived extracellular-vesicle product. According to the manufacturer, the product contains extracellular vesicles and soluble paracrine factors derived from donated perinatal tissue [9].

Because detailed independent biochemical and lot-specific characterization was not available in the retrospective clinical dataset, PPX™ and DayZero™ were not assumed to be biologically equivalent. For the purposes of this report, they are collectively referred to as extracellular-vesicle preparations. This terminology does not constitute independent confirmation of product composition, purity, or compliance with the minimal information for studies of extracellular vesicles criteria [3].

Outcome measures

KOOS-JR was used to quantify knee pain, stiffness, and functional limitation. This validated seven-item questionnaire is transformed into an interval score ranging from 0 to 100, with higher values representing better knee health [10].

The primary outcome was the paired change in KOOS-JR from the pre-injection baseline to six weeks after treatment. Secondary outcomes included the paired change from baseline to the latest available numeric KOOS-JR, the proportion of patients demonstrating any improvement in KOOS-JR, subsequent nonsurgical intervention, subsequent TKA, and documented administration-related adverse events.

Subsequent TKA was recorded as a descriptive clinical event rather than classified as treatment failure. Patients’ observed KOOS-JR values were retained in the patient-reported outcome analyses. Because follow-up duration was nonuniform and exact dates and indications for subsequent TKA were not consistently available, the analysis does not estimate time-dependent arthroplasty risk or treatment failure.

Safety assessment

Adverse events were identified retrospectively through review of the available practice medical record, including office notes, telephone encounters, patient portal communications, and emergency department, hospital, or other external records when available within the practice chart. Events of interest included documented injection-site reactions, severe post-injection pain or swelling, synovitis, infection, allergic or hypersensitivity reactions, thromboembolic events, hospitalization, and other systemic events considered potentially related to treatment. No systematic prospective adverse-event surveillance was performed, and records from care obtained outside the practice were available only when incorporated into the practice medical record. Accordingly, this analysis describes documented administration-related adverse events and should not be interpreted as a comprehensive assessment of treatment safety.

Statistical analysis

Continuous variables were summarized using means and standard deviations or medians and ranges, as appropriate. Categorical variables were reported as frequencies and percentages. Paired changes in KOOS-JR were assessed using a paired-samples t-test. A Wilcoxon signed-rank test was also performed as a distribution-robust sensitivity analysis.

For the primary patient-level analysis, one eligible knee per patient was included to preserve independence of observations. For patients with separate treatment records for both knees, the earliest qualifying knee treatment was designated as the index knee. Records describing bilateral knee treatment with only a single KOOS-JR score that could not be attributed to a specific knee were excluded from knee-specific patient-reported outcome analyses. Inclusion in each paired analysis required an attributable numeric baseline KOOS-JR score and an attributable numeric score at the corresponding follow-up interval. Missing values were not imputed.

No prospective sample-size calculation was performed because of the retrospective and exploratory study design. No adjustment was made for multiple secondary comparisons. All analyses were performed using Python 3 (Python Software Foundation, Fredericksburg, VA, USA) and the SciPy scientific computing library (NumFOCUS, Austin, TX, USA).

## Results

Cohort characteristics

The patient-level cohort included 47 unique patients (Table 1). The mean age was 74.6 years (SD, 12.7; range, 44-94 years), and 28 (59.6%) patients were female. The cohort comprised 51 treated knees: 43 patients contributed one treated knee, and four contributed two treated knees. Seven (13.7%) knees received PPX™, and 44 (86.3%) received DayZero™.

**Table 1:** Changes in KOOS-JR scores following intra-articular exosome-based treatment The primary analysis included one eligible index knee per patient to preserve independence of observations. Records in which a single KOOS-JR score could not be attributed to a specific knee following bilateral treatment were excluded from knee-specific patient-reported outcome analyses. Patients who subsequently underwent TKA were retained in the paired analyses when eligible KOOS-JR scores were available. Missing values were not imputed. CI, confidence interval; KOOS-JR, Knee injury and Osteoarthritis Outcome Score for Joint Replacement; SD, standard deviation; TKA, total knee arthroplasty.

KOOS-JR comparison	Paired observations	Baseline, mean ± SD	Follow-up, mean ± SD	Mean change (95% CI)	Paired t-test	Wilcoxon test
Baseline to six weeks	33	60.0 ± 8.0	78.5 ± 13.8	+18.5 (13.2-23.8)	t(32) = 7.14; p<0.001	p<0.001
Baseline to latest follow-up	36	60.6 ± 10.6	77.4 ± 13.3	+16.8 (11.4-22.3)	t(35) = 6.30; p<0.001	p<0.001

Complete, laterality-attributable paired baseline and six-week KOOS-JR scores were available for 33 patients in the primary analysis. Mean KOOS-JR increased from 60.0 at baseline to 78.5 at six weeks, corresponding to a mean improvement of 18.5 points (95% CI, 13.2-23.8). The difference was statistically significant on the paired-samples t-test, t(32) = 7.14, p<0.001, with a consistent result on the Wilcoxon signed-rank sensitivity analysis (p<0.001). Thirty (90.9%) patients improved, and three (9.1%) worsened; 20 of 33 (60.6%) patients demonstrated an improvement of at least 14 points.

Complete, laterality-attributable paired baseline and latest follow-up KOOS-JR scores were available for 36 patients. Mean KOOS-JR increased from 60.6 at baseline to 77.4 at latest follow-up, corresponding to a mean improvement of 16.8 points (95% CI, 11.4-22.3). The difference was statistically significant on the paired-samples t-test, t(35) = 6.30, p<0.001, with a consistent result on the Wilcoxon signed-rank sensitivity analysis (p<0.001). Thirty (83.3%) patients improved, two (5.6%) were unchanged, and four (11.1%) worsened; 21 of 36 (58.3%) patients demonstrated an improvement of at least 14 points.

Patients who subsequently underwent TKA were retained in the patient-reported outcome analyses when eligible KOOS-JR scores were available. Subsequent TKA was analyzed separately as a descriptive clinical event.

Thirty of 33 (90.9%) patients had higher KOOS-JR scores at six weeks than at baseline (Figures 1, 2).

**Figure 1:**
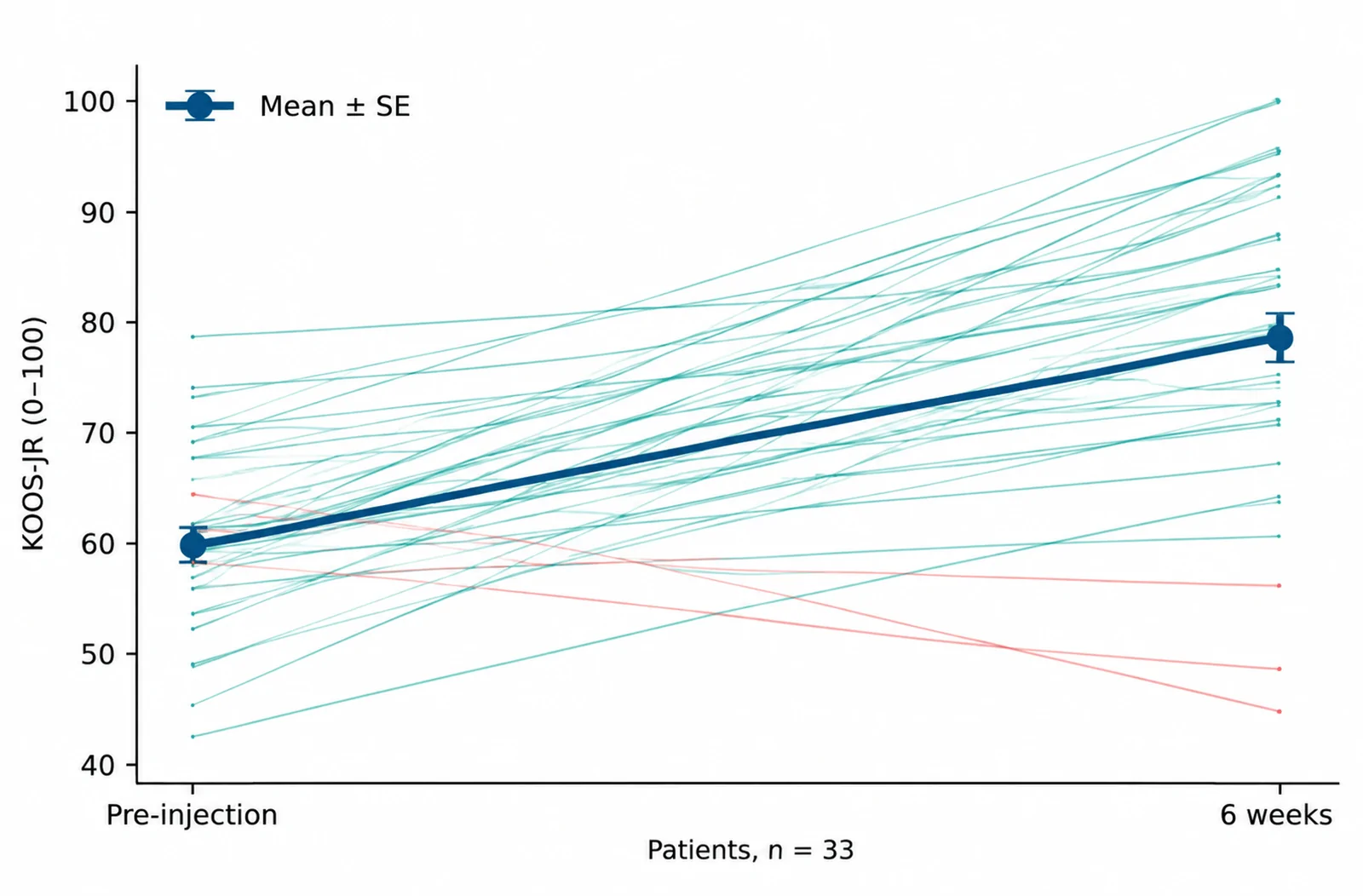
Paired patient-level KOOS-JR scores before injection and at six weeks Individual lines represent the 33 patients with complete, laterality-attributable paired baseline and six-week data included in the primary analysis; one index knee per patient was included. The heavy line and error bars represent the mean ± standard error. KOOS-JR, Knee injury and Osteoarthritis Outcome Score for Joint Replacement; SE, standard error.

**Figure 2:**
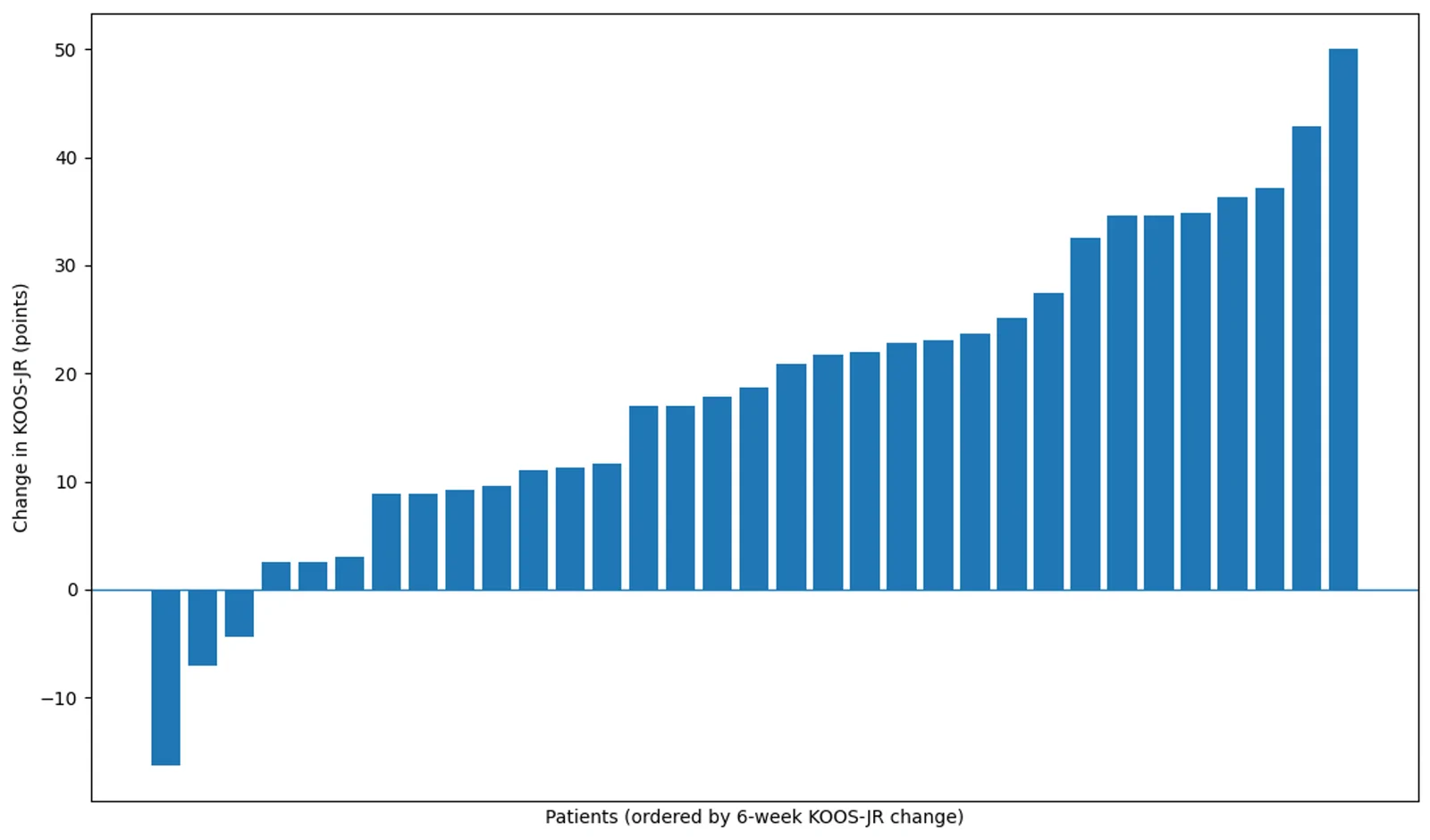
Waterfall plot of individual six-week KOOS-JR change Patient-level distribution of individual six-week KOOS-JR change among the 33 patients in the primary paired analysis. Bars represent the change from baseline to six weeks for each patient and are ordered from the lowest to highest change. Positive values indicate higher KOOS-JR scores at six weeks than at baseline. One index knee per patient was included in the analysis. KOOS-JR, Knee injury and Osteoarthritis Outcome Score for Joint Replacement.

The mean change exceeded a recently reported anchor-based KOOS-JR minimal clinically important difference of approximately 16 points after TKA [10]. That threshold was derived in a surgical cohort and has not been validated for intra-articular exosome therapy.

Among 36 patients with complete, laterality-attributable paired baseline and latest available KOOS-JR scores, mean KOOS-JR increased from 60.6 (SD, 10.6) to 77.4 (SD, 13.3), corresponding to a mean paired increase of 16.8 points (95% CI, 11.4-22.3; p<0.001). Thirty of 36 patients (83.3%) demonstrated improvement, two (5.6%) were unchanged, and four (11.1%) demonstrated worsening scores. Interpretation of this later endpoint is limited by heterogeneous follow-up intervals and intervening treatments.

Subsequent treatment, TKA conversion, and safety

At latest documented follow-up, 28 patients (59.6%) had no documented additional treatment, 16 (34.0%) underwent additional nonsurgical treatment, and 3 (6.4%) subsequently underwent TKA (Figure 3).

**Figure 3:**
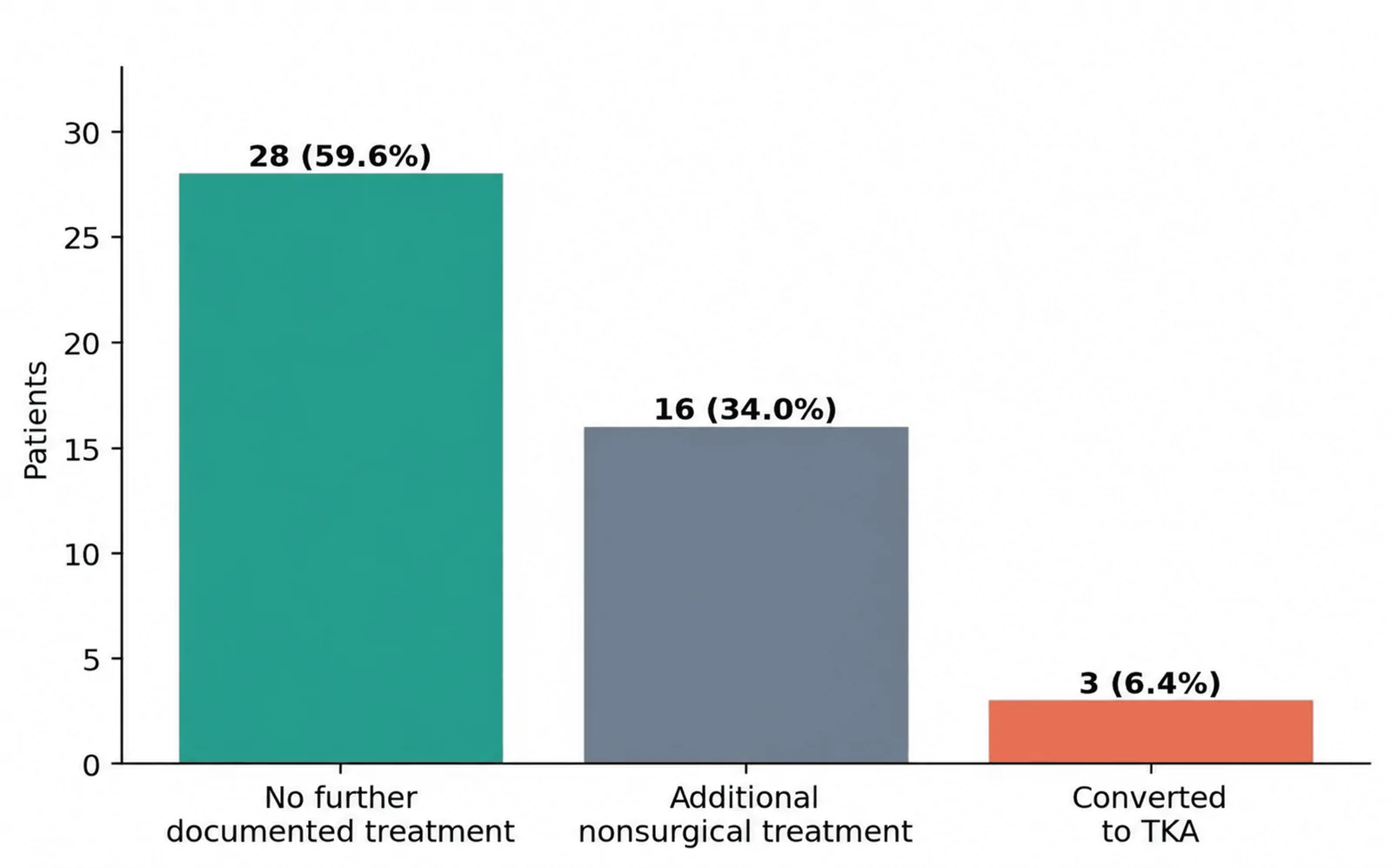
Clinical disposition at latest documented follow-up Subsequent TKA is shown as a descriptive clinical event rather than a treatment failure. Follow-up duration was nonuniform. TKA, total knee arthroplasty.

Subsequent TKA was recorded as a descriptive clinical event rather than treatment failure. Because follow-up duration was nonuniform and exact arthroplasty dates and standardized indications were not consistently available, these data do not estimate time-dependent arthroplasty risk. No administration-related adverse events were documented in the available practice records. Because adverse-event ascertainment was retrospective and care obtained outside the practice may not have been captured, this finding should not be interpreted as establishing treatment safety.

## Discussion

The principal finding of this retrospective series was that, among 33 patients with complete, laterality-attributable paired baseline and six-week KOOS-JR data, mean KOOS-JR increased by 18.5 points (95% CI, 13.2-23.8), with 30 of 33 (90.9%) patients demonstrating higher scores at six weeks. At the latest follow-up, 36 patients with complete paired data demonstrated a mean increase of 16.8 points (95% CI, 11.4-22.3), with 30 of 36 patients (83.3%) demonstrating higher scores. Interpretation of the latest-follow-up findings is limited by nonuniform follow-up duration and subsequent interventions, including corticosteroid or hyaluronic acid injections, cryoneurolysis, and surgery. Three patients subsequently underwent TKA; these events were recorded descriptively and did not alter or replace their observed KOOS-JR values.

The present findings should be interpreted in the context of the randomized, triple-blind, placebo-controlled trial by Bolandnazar et al., which evaluated intra-articular placental mesenchymal stromal cell-derived extracellular vesicles in patients with knee osteoarthritis [7]. Despite treatment administration, the investigators found no statistically significant differences between extracellular-vesicle-treated knees and saline-injected control knees in pain, functional outcomes, or MRI findings at two and six months. Although the present study demonstrated a statistically significant mean improvement in KOOS-JR at six weeks, its retrospective, uncontrolled design cannot distinguish a treatment-specific effect from placebo responses, regression to the mean, or natural symptom fluctuations. Differences in patient populations, extracellular-vesicle preparations, outcome measures, and assessment intervals further limit direct comparisons between studies. Importantly, the negative findings of the randomized trial emphasize that improvement from baseline alone does not establish therapeutic efficacy. Accordingly, the improvements observed in the present series should be considered descriptive and hypothesis-generating rather than evidence of a causal treatment benefit.

Comparison with other knee OA treatments

Direct comparison across studies is inherently limited by different populations, follow-up intervals, comparators, and outcome instruments. In randomized and network meta-analytic evidence, intra-articular corticosteroid generally provides short-term symptom relief, while hyaluronic acid has a later onset and modest average benefit; PRP frequently ranks better than corticosteroid or hyaluronic acid at 3-12 months, although protocols remain heterogeneous [1,2,11-13]. Most injection trials report WOMAC, IKDC, or VAS rather than KOOS-JR, so their numerical changes cannot be validly equated with the present KOOS-JR change. The current uncontrolled six-week improvement is therefore best interpreted as a signal requiring randomized confirmation, not evidence of superiority.

TKA remains the most reliable treatment for end-stage symptomatic knee OA. In a recently published single-surgeon cohort of 200 manually instrumented TKAs, mean KOOS-JR improved from 56.17 preoperatively to 78.32 at six weeks (change 22.15) and 89.4 at one year (change 33.23) [14]. The present cohort demonstrated a mean 18.5-point increase in KOOS-JR at six weeks in the primary patient-level analysis. This contextual comparison should not be interpreted as evidence of equivalence or superiority: the cohorts were not randomized or adjusted, disease severity and indications likely differed, and injection does not provide the structural correction or established durability of arthroplasty. A contextual comparison of the current findings with commonly used treatments for symptomatic knee osteoarthritis, including their reported outcomes, treatment windows, and safety considerations, is presented in Table 2.

**Table 2:** Contextual comparison with common treatments for symptomatic knee osteoarthritis KOOS-JR, Knee injury and Osteoarthritis Outcome Score for Joint Replacement; PROM, patient-reported outcome measure.

Treatment	Typical evidence window	Outcome evidence	Safety/burden	Interpretation vs current cohort
Extracellular vesicle injection (current cohort)	6 weeks; variable latest follow-up	KOOS-JR +18.5 at six weeks; +16.8 at latest follow-up	0 documented administration-related complications; investigational product	Promising uncontrolled signal; efficacy not established
Intra-articular corticosteroid	Days to several weeks	Short-term pain/function benefit; wanes over time [1,2]	Low procedure burden; repeated use has limitations	No valid direct numerical comparison because instruments differ
Hyaluronic acid	4 weeks to 6 months	Modest/variable benefit; guideline recommendations differ [1,2,11-13]	Injection-related reactions possible	No valid direct numerical comparison because instruments differ
Platelet-rich plasma	3-12 months	Often superior to hyaluronic acid/corticosteroid in meta-analyses, with protocol heterogeneity [11-13]	Autologous injection; transient pain/swelling possible	Requires head-to-head trial using same PROM
Total knee arthroplasty	6 weeks to 1 year and beyond	Published KOOS-JR +22.15 at 6 weeks and +33.23 at 1 year [14]	Surgical recovery and recognized perioperative risks	Established definitive option; contextual, nonrandomized comparison only

Safety and interpretation of exosome terminology

The absence of documented complications is clinically important and supports short-term procedural tolerability in this series. However, zero observed events in 47 patients does not establish safety; the approximate exact 95% upper confidence limit is 7.5%, and retrospective records may miss transient or externally managed events. Furthermore, product composition, potency, sterility testing, and EV characterization materially influence safety and biologic interpretation. MISEV guidance recommends precise reporting and discourages assigning the term exosome without evidence of endosomal origin [3]. Regulatory agencies have also warned that exosome products marketed for orthopedic conditions are not FDA-approved [8].

Limitations

This study has several limitations. First, its retrospective, uncontrolled design precludes causal attribution of observed changes in KOOS-JR to the extracellular-vesicle intervention. Selection bias, expectancy and placebo effects, regression to the mean, and the natural variability of OA symptoms may have contributed to the observed changes. No untreated, sham-injection, placebo, corticosteroid, hyaluronic acid, PRP, or standard-care comparison group was available.

Second, baseline and follow-up KOOS-JR data were incomplete, and follow-up duration was nonuniform. Later KOOS-JR measurements may also have been influenced by subsequent treatments, including corticosteroid or hyaluronic acid injections, cryoneurolysis, and surgery. Accordingly, longer-term changes cannot be attributed specifically to the index extracellular-vesicle injection.

Third, two biologically distinct extracellular-vesicle preparations were included and pooled for the primary analysis. PPX™ is an autologous platelet-derived preparation, whereas DayZero™ is an allogeneic perinatal tissue-derived preparation. Product selection was based on patient preference and was not randomized; therefore, unmeasured differences between patients selecting each product may have introduced confounding. The marked imbalance in product use (7 PPX™ and 44 DayZero™ treated knees) also limits meaningful comparative analysis between products. Independent biochemical and lot-specific characterization of the administered preparations was not available.

Fourth, several potentially important clinical variables, including radiographic OA severity, symptom duration, prior treatment history, body mass index, comorbidities, analgesic use, and standardized physical therapy exposure, were not consistently available. These factors could influence both patient-reported outcomes and subsequent treatment decisions.

Fifth, KOOS-JR is a validated patient-reported outcome measure for knee OA and arthroplasty populations, but its measurement properties and thresholds for clinically important change have not been specifically established for intra-articular extracellular-vesicle treatment. Accordingly, the prespecified ≥14-point improvement threshold was used descriptively and should not be interpreted as establishing a clinically meaningful treatment response in this setting.

Sixth, some patients underwent treatment of both knees. To preserve independence in the primary analysis, one index knee per patient was included, and KOOS-JR observations that could not be attributed to a specific knee were excluded. This approach reduced the available analytic sample and may have introduced additional selection effects.

Seventh, adverse events were identified retrospectively from documentation available within the practice medical record rather than through systematic prospective surveillance. Care obtained outside the practice may not have been captured unless corresponding records were incorporated into the practice chart. Therefore, the absence of documented administration-related adverse events should not be interpreted as evidence establishing treatment safety.

Eighth, subsequent TKA was recorded as a descriptive clinical event. Follow-up duration was nonuniform, and exact dates and standardized indications for arthroplasty were not consistently available. Consequently, time-to-event or survival analysis could not be performed, and the observed proportion undergoing TKA should not be interpreted as an estimate of time-dependent arthroplasty risk.

Finally, treatment was provided in a private clinical practice and was paid for by patients, generating revenue for the practice. This represents a potential financial conflict of interest and potential source of selection bias. KOOS-JR scores were patient-reported outcomes and were analyzed as recorded in the clinical record; treatment decisions and subsequent interventions were based on routine clinical care rather than study assignment.

## Conclusions

In this retrospective single-practice case series, mean KOOS-JR was 18.5 points higher at six weeks than at baseline among 33 patients with complete, laterality-attributable paired data following intra-articular administration of extracellular-vesicle products. Later outcome assessments were limited by nonuniform follow-up and subsequent treatments. Three of 47 patients subsequently underwent TKA. No administration-related adverse events were documented in the available practice records; however, retrospective ascertainment and the limited sample size preclude conclusions regarding safety or tolerability. These descriptive findings do not establish efficacy, clinical importance, comparative effectiveness, structural disease modification, or long-term benefit. Prospective controlled studies using well-characterized products, standardized follow-up, prespecified management of subsequent interventions, and active adverse-event surveillance are warranted.
